# Proteogenomic Identification of a Novel Protein-Encoding Gene in Bovine Herpesvirus 1 That Is Expressed during Productive Infection

**DOI:** 10.3390/v10090499

**Published:** 2018-09-14

**Authors:** Victoria A. Jefferson, Kaley A. Barber, Fouad S. El-mayet, Clinton Jones, Bindu Nanduri, Florencia Meyer

**Affiliations:** 1 Department of Biochemistry, Molecular Biology, Entomology & Plant Pathology, Mississippi State University, 32 Creelman St., Starkville, MS 39762, USA; vaj13@msstate.edu (V.A.J); kaleyabarber@gmail.com (K.A.B.); 2 Department of Veterinary Pathobiology, Center for Veterinary Health Sciences, Oklahoma State University, Stillwater, OK 74078, USA; fouad.elmayet@okstate.edu; 3 Department of Virology, Faculty of Veterinary Medicine, Benha University, Moshtohor 13736, Kaliobyia, Egypt; clint.jones10@okstate.edu; 4 Department of Basic Sciences at the College of Veterinary Medicine, Mississippi State University, 240 Wise Center Drive, Starkville, MS 39762, USA; bnanduri@cvm.msstate.edu

**Keywords:** Bovine herpesvirus, proteogenomic mapping, gene discovery

## Abstract

Bovine herpesvirus 1 (BoHV-1) is one of several microbes that contributes to the development of the bovine respiratory disease (BRD) and can also induce abortions in cattle. As other alpha-*herpesvirinae* subfamily members, BoHV-1 efficiently replicates in many cell types and subsequently establishes a life-long latent infection in sensory neurons. BoHV-1 encodes more than 70 proteins that are expressed in a well-defined manner during productive infection. However, in silico open reading frame (ORF) prediction of the BoHV-1 genome suggests that the virus may encode more than one hundred proteins. In this study we used mass spectrometry followed by proteogenomic mapping to reveal the existence of 92 peptides that map to previously un-annotated regions of the viral genome. Twenty-one of the newly termed “intergenic peptides” were predicted to have a viable ORF around them. Twelve of these produced an mRNA transcript as demonstrated by strand-specific RT-PCR. We further characterized the 5′ and 3′ termini of one mRNA transcript, ORF-A, and detected a 55 kDa protein produced during active infection using a custom-synthesized antibody. We conclude that the coding potential of BoHV-1 is underestimated.

## 1. Introduction

Bovine herpesvirus type 1 (BoHV-1) is a linear, double stranded DNA virus composed of a host-derived lipid envelope and tegument proteins surrounding an icosahedral nucleocapsid. It is a member of the alphaherpesvirus subfamily within the *Varicellovirus* genus [1]. Three subtypes, BoHV-1.1, BoHV-1.2a, and BoHV-1.2b are recognized and are distinguished by their differences in endonuclease restriction sites [2,3]. BoHV-1.1 is commonly found in respiratory infections in feedlot cattle, while genital infections and abortions are more common in breeding cattle [4]. In fact, BoHV-1 is the most frequently diagnosed cause of viral abortion in North America. The subtype 2a typically causes balanopostitis and abortions and is not usually associated with respiratory disease, while subtype 2b can be associated with respiratory disease but not abortions [5,6].

BoHV-1 and other viruses such as bovine respiratory syncytial virus (BRSV) or bovine parainfluenza virus type 3 (bPI3V), are attributed to host immune suppression and a condition known as bovine respiratory disease (BRD) in which the attenuated immune response increases the risk of secondary infections of bacteria like *Mannheimia haemolytica*, *Pasteurella multocida*, or *Histophilus somni*, which can then lead to pneumonia [7,8,9]. A BoHV-1 entry protein encoded by the poliovirus receptor related 1 gene is a known marker of BRD susceptibility in Holstein calves, confirming that BoHV-1 is an important BRD cofactor [10].

Host immunosuppression by BoHV-1 can be partially attributed to the ability of the virus to reduce the number of CD4^+^ T lymphocytes, induce apoptosis in lymphoid tissues, and downregulate the number of MHC class I molecules [11,12]. In addition, BoHV-1 can establish multiple acute infections throughout life due to its ability to establish latency in the sensory neurons of the trigeminal ganglia until the virus is reactivated by stress or immune suppression [4,13]. Due to its ability to cause widespread infection, BoHV-1 has a persistent negative economic impact on the cattle industry [14,15]. Essential to creating an effective vaccine are the understanding of viral mechanisms necessary for productive infection and immune stimulation, and identification of viral and host proteins that participate in these processes.

BoHV-1 genome sequencing and structural annotation (demarcation of the boundaries of coding elements) was conducted internationally in 1996 by a collaboration of several groups that analyzed genome segments created by the Hind III restriction endonuclease [16,17,18,19,20]. When it was completely sequenced, the nearly 140 kb BoHV-1 genome, which is divided into a 104 kb unique long (U_L_) and a 10 kb unique short (U_S_) region, was found to have 67 unique open reading frames (ORFs) and two ORF duplications, encoded on both strands in one of six different reading frames [21]. However, this sequence was essentially a chimera of multiple strains of the BoHV 1.1 and 1.2 subtype. The Cooper strain of the virus was sequenced in 2013, providing access to a full sequence for the subtype 1.1 [22]. Even though the differences between the BoHV-1.1 sequence and the (old) composite sequence were not extensive, the original annotation was not modified. Therefore, a more accurate knowledge of the viral coding capacity could be achieved through re-annotation of the BoHV-1.1 sequence.

Genome structural annotation is an integral part of genome sequencing efforts and is often accomplished by computational gene prediction based on comparative genomics. Computational gene prediction is performed on a finished genome sequence by searching databases for sequence similarities, or by analyzing multiple genomes at once to characterize similar elements [23,24]. Proteogenomic mapping (PGM) has emerged as a valuable complementary genome structural annotation method. PGM approach involves mass spectrometry-based identification of all expressed peptides from a biological sample, mapping the peptides to the genome sequence to identify genome coordinates to improve existing annotation and to identify novel protein-encoding ORFs that were missed by the computational prediction algorithms [25,26]. This experiment-based approach to genome structural annotation can capture context dependent expression of the genome and generate a more accurate genomic map, and there is increasing appreciation for the need to combine experimental genome annotation approaches with computational predictions for comprehensive annotation of genome sequences.

The proteogenomic mapping has been beneficial in annotating the genome of herpesviruses and identifying post-translational modifications of viral proteins [25,27,28,29,30]. They have been utilized in the case of many herpesviruses for annotation, characterization, and discovery of novel protein-coding genes. MS/MS has been essential for the characterization of proteins produced by Kaposi’s sarcoma herpesvirus (KSHV) coding capacity [27,31]. Proteomics has also been exceedingly useful in the protein characterization of non-human gammaherpesviruses such as rhesus monkey rhadinovirus, murid herpesvirus 4, and bovine herpesvirus 4 [30,32,33] Proteogenomic mapping made possible the identification of proteins produced by channel catfish virus during infection [25]. Many of the same methods have helped clarify the genomes of several avian herpesviruses [34].

In the present study, we used proteogenomic mapping to identify viral proteins expressed during infection. Our results confirmed the expression of 57 previously annotated viral proteins as well as identified many peptides that mapped to genomic coordinates of BoHV-1.1 Cooper strain with no annotated ORFs. We further characterized one of these novel genes named “ORF-A”, with respect to mRNA transcript boundaries and its RNA and protein expression. Our results suggest that the coding potential of BoHV-1 is underestimated in the current computational-based annotation.

## 2. Materials and Methods

### 2.1. Cells and Virus

Madin Darby Bovine Kidney (MDBK) and cells resistant to infection by the bovine viral diarrhea virus (CRIB-1) were cultured in Dulbecco’s modified Eagle’s medium (DMEM) supplemented with 5% (*v*/*v*) fetal calf serum, 100 U/mL penicillin and 100 μg/mL streptomycin. CRIB-1 cells are derived from MDBK cells [35]. Rabbit skin (RS) cells were grown in minimal essential medium (MEM) supplemented with 10% (*v*/*v*) FCS, penicillin (10 U/mL), and streptomycin (100 μg/mL). All cells were kept at 37 °C in a humidified incubator with 5% CO_2_ supply. The Bovine herpesvirus 1.1 (BoHV-1), Cooper isolate (GenBank Accession number JX898220.1) was used to infect cultured cells at multiplicity of infection (MOI) of 1 or 5.

### 2.2. Infected Whole Cell Sample Preparation

CRIB-1 cells were infected at a multiplicity of infection (MOI) of 5 and incubated at 4 °C for one hour to synchronize infection, before placing in humidified CO_2_ chamber at 37 °C. Infected samples were collected at 2, 8, and 16 h post infection (hpi). Cells were washed with cold phosphate-buffered saline (PBS), scraped and centrifuged for 30 min at 4 °C, and stored at −80 °C until processing for mass spectrometry. Whole cell extracts from uninfected and BoHV-1-infected samples (*n* = 3 for each) were solubilized in 10% SDS (to a final concentration of 4%) and treated with protease inhibitor cocktail (1/10 of final sample volume) (Sigma-Aldrich, Saint Louis, MO, USA). Samples were prepared for quantification with the Thermo Scientific Pierce BCA Protein Assay Kit (Fisher Scientific, Pittsburgh, PA, USA) and protein concentration was determined using a plate spectrophotometer (ThermoMax Microplate, Molecular Devices, Sunnyvale, CA, USA). Samples containing 100 µg protein were precipitated with methanol and chloroform (4:1), washed with methanol, spun and air-dried in a desiccator prior to tryptic digestion. Protein samples were treated with 8 M urea (RT for 30 min), reduced (0.005 M DTT at 65 °C for 10 min) and alkylated (0.01 M iodoacetamide at 37 °C for 30 min). Protein samples were diluted with water, pH was adjusted to 7.5 and were digested with molecular biology grade porcine trypsin (2 µg at 37 °C, overnight, 50:1 ratio of protein:trypsin, Promega Corporation, Madison, WI, USA). Tryptic peptides were cleaned up using a strong cation exchange (SCX) trap (Michrom BioResources Inc., Auburn, CA, USA), eluted in high salt buffer (0.005 M NaH_2_ PO_4_, 25% acetonitrile, 0.25 M KCl, pH 3) and dried. Dried tryptic peptides were desalted using a peptide macrotrap (Michrom BioResources Inc., Auburn, CA, USA), eluted in 0.1% triflouroacetic acid, 95% acetonitrile solution, and air-dried in a desiccator.

### 2.3. Liquid Chromatography-Tandem Mass Spectrometry

Spectral data were collected using an Orbitrap LTQ Velos mass spectrometer (Thermo Fisher Scientific, Waltham, MA, USA) linked with an UltiMate 3000 nano flow HPLC system (Thermo Fisher Scientific, Waltham, MA, USA). Two micrograms of protein tryptic digest were loaded on reversed phase fused silica Acclaim PepMap C18 column (75 µm × 150 mm, Thermo Fisher Scientific). A constant flow rate of 0.3 microliters per minute was used to separate and elute peptides with a 60-min long linear gradient of acetonitrile in 0.1% formic acid: 2–55% for 35 min, 95% for 10 min, 2% for 15 min. Peptides were detected by linear trap mass detector, in data dependent acquisition (DDA) mode with dynamic exclusion. One MS scan event (*m*/*z* range: 300–2000) was followed by seven tandem mass spectrometry (MS/MS) scans for the seven most intense ions detected in MS scan. Set parameters included: Normalized collision energy: 35%, AGC (automatic gain control) “on” with MSn Target 4 × 10^4^, isolation width (*m*/*z*): 1.5, capillary temperature 170 °C, spray voltage 1.97 kV.

The raw files were searched using the SEQUEST algorithm of the Proteome Discoverer 1.1 software (Thermo Fisher Scientific) with given parameters: Lowest and highest charge: +1 and +3, respectively; minimum and maximum precursor mass: 300 and 6000 Da, respectively; minimum S/N ratio: 3; enzyme: trypsin; maximum missed cleavages: 2, FDR = 0.01; dynamic modifications: cysteine carbamidomethylation (+57.021), methionine oxidation (+15.995), methionine dioxidation (+31.990).

### 2.4. Proteogenomic Mapping

The proteogenomic target database was created by using the EMBOSS tool suite [36] to translate the entire BoHV-1.1 genome (Accession: JX898220.1) in all six reading frames, mask all stop codons, and split the resulting amino acid sequences into pseudo-proteins of length 500 overlapping by 50 amino acids. The decoy database was created by randomizing the sequences in the target database. The spectra were searched against both databases using X!tandem and OMSSA [37]. The X!tandem and OMSSA results were combined and filtered using the Proteomics Toolset (sf.net/projects/proteomicstools/). For each time point, the filter was optimized for a peptide FDR less than 5%. The peptides that passed filtering were aligned to the genome using tblastn [38]. Any alignment without 100% identity over the entire peptide was removed from further analysis. In addition, this list of peptides was blasted to reveal no matches against all host Bos Taurus proteins currently on NCBI. The bedtools tool suite was used to remove any alignment that overlapped the currently annotated ORFs, leaving only the intergenic peptides [39]. The genomic sequence corresponding to novel peptides was scanned up- and downstream in search of start and stop codons that constitute the boundaries of a potentially new gene [26]. In this study we define unique peptides as peptides mapping to a single location in the genome.

### 2.5. RNA Extraction, RT-PCR, and RACE

Messenger RNA (mRNA) was extracted from CRIB-1 cells that were mock infected or infected for 16 h with a MOI of 5 using the magnetic mRNA isolation kit (#S1550S, New England Biolabs, Ipswich, MA, USA). This was followed by DNase I treatment (#EN0521, Thermo Fischer Scientific), and the synthesis of complementary DNA (cDNA) via reverse transcriptase using the Invitrogen Superscript III module (#18080-044, Carlsbad, CA, USA) and *gene specific* primers produced by Eurofins (Louisville, KY, USA; see Table 1a). For the controls, cDNA was synthesized using random hexamer primers provided in the Invitrogen module. The strand-specific cDNA was used as a template for polymerase chain reaction (PCR) using the OneTaq module (#M0480L, New England Biolabs).

Rapid amplification of 3′ and 5′ cDNA ends (RACE) was performed using the Invitrogen GeneRacer Kit according to manufacturer’s instructions (L1502-01) and gene-specific primers (Eurofins; see Table 1b). Touchdown PCR was performed for RACE to increase the specificity of the transcript being amplified. To elucidate the 3′ end of the transcript, reverse primers designed in a stepwise manner were used in conjunction to the original “A” forward primer eventually capturing its full length. Transcription profiling was carried out at 4, 8, 12, 15, and 24 hpi via RT-PCR using reverse primer 9. Negative controls were conducted by using the DNase treated mRNA as a template instead of the cDNA to discard the possibility of genomic DNA contamination.

### 2.6. DNA Gel Electrophoresis, Purification, and Sequencing

PCR amplicons were visualized using a 1% agarose gel in TAE buffer (40 mM Tris (pH 7.6), 20 mM acetic acid, and 1 mM EDTA). Bands for the 5′ RACE and or other amplicons were excised with a clean scalpel and purified using the Monarch nucleic acid purification kit (#T1020, New England Biolabs). The purified DNA was quantified using NanoDrop, sent to Eurofins for sequencing, and compared to the full BoHV-1 Cooper sequence (accession: JX898220.1).

### 2.7. Generation of Peptide Specific Antiserum Directed against ORF-A

Two peptides present in ORF-A (RPARRGGHRRGEIGDA and DQPPKVAGRSTKHVGRGS) were used to generate peptide specific antiserum in rabbits (Pierce Biotechnology, Waltham, MA, USA). The antibody was generated by mixing both peptides in equal molar concentrations prior to injection of rabbits. All injections were performed with both peptides. The serum was collected, and peptide specific antibodies purified by affinity chromatography.

### 2.8. Western Blot Analysis

For Western blot studies, the designated cell line was infected with a MOI of 1. At different times after infection, cell cultures were washed with PBS and suspended in RIPA lysis buffer (50 mM Tris HCL (pH 8.0), 150 mM NaCL, 2 mM EDTA (pH 8.0), 1% NP-40, 0.5% sodium deoxycholate, 0.1% SDS) and one tablet of Complete Protease Inhibitor (Roche Molecular Biochemicals, Mannheim, Germany) in 10 mL of buffer. Cell lysate was incubated on ice for 30 min, sonicated, and then clarified by centrifugation at 13,000 RPM at 4 °C for 15 min. Protein concentrations were determined by the Bradford assay (Bio-Rad, Hercules, CA, USA). For SDS-PAGE, proteins were mixed with an equal amount of 2× sample loading buffer (62.5 mM Tris-HCl (pH 6.8), 2% SDS, 50 mM dithiothreitol, 0.1% bromophenol blue, 10% glycerol) and boiled for 5 min. Proteins were separated in an SDS–10% PAGE gel.

After electrophoresis, proteins were transferred onto a polyvinylidene difluoride membrane (Immobilon-P; Millipore, Burlington, MA, USA) and blocked for 1 h in 5% *w*/*v* nonfat dry milk with 1× Tris-buffered saline–0.1% Tween 20 (TBS-T). Membranes were then incubated with the designated primary antibody at 4 °C with gentle shaking overnight. The ORF-A specific antibody was diluted 1:1000 in the blocking solution. An antibody directed against tubulin was used as a loading control. After 45 min of washing with TBS-T, the blots were incubated with secondary antibodies (peroxidase-conjugated immunoglobulin G (Amersham Biosciences, Little Chalfont, UK), which was diluted 1:2000 in 5% nonfat milk in TBS-T for 1 h. Blots were washed 45 min with TBS-T and exposed to Amersham ECL reagents, and then autoradiography was performed. The secondary donkey anti-rabbit antibody (NA9340V, Chicago, IL, USA) was purchased from GE Healthcare.

## 3. Results

### 3.1. Proteogenomic Analysis of Infected Cells Identified Potential Novel Open Reading Frames

Guided by the hypothesis that the coding potential of BoHV-1 was underestimated, we conducted an ORF prediction analysis of the BoHV-1 genome using the FGENESV algorithm (www.softberry.com). This algorithm for viral gene prediction identified 116 potential ORFs in the 2012 sequence corresponding to the Cooper strain (JX898220.1 Bovine herpesvirus type 1.1 isolate NVSL challenge 97-11). The same analysis done using the 1996 multi-strain BoHV-1 sequence (NC_001847.1 Bovine herpesvirus 1) yielded 109 potential ORFs. Both these analyses revealed a significantly higher number of ORFs than the originally annotated 69 ORFs. In addition, our analysis revealed slight differences ORF boundaries for about 10 of the currently annotated ORFs (data not shown). Our computational analysis of both old and new BoHV-1 genomes suggested that the coding potential of the BoHV-1 genome is underestimated and constituted the starting point for exploring the genome with a new approach that incorporates experimental data into the re-annotation process.

**Figure 1:**
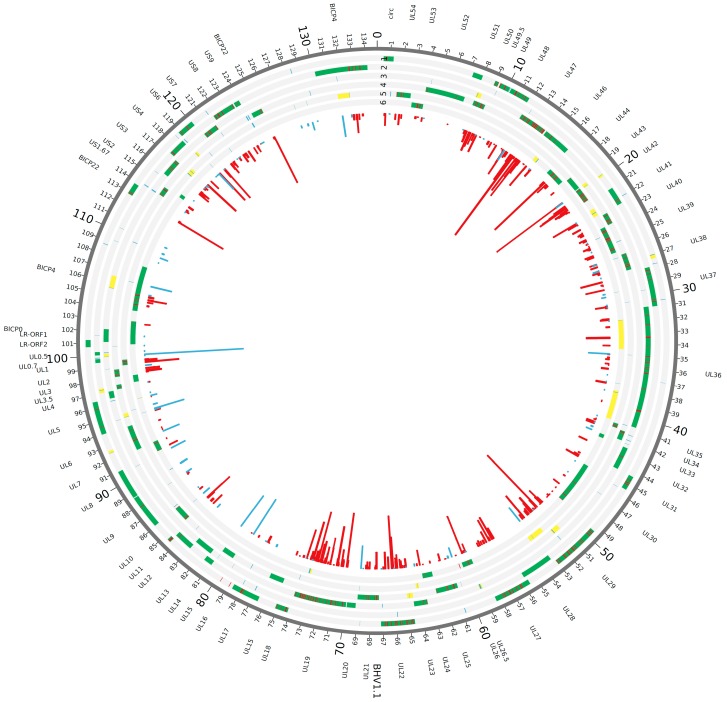
MS/MS peptides mapped to the BoHV-1 genome. The 134 kb genome is displayed in the six possible reading frames (six different tracks). Currently annotated viral ORFs are shown in green in their corresponding reading frame; gene names are shown on the outside of the circle. MS/MS spectra are shown both as a histogram in the center as well as mapped onto the corresponding reading frame. Spectra mapping to coding regions are shown in red, while intergenic spectra (refer to text for explanation) appear in blue. Twenty-one viable ORFs surrounding intergenic spectra are shown in yellow in the corresponding reading frame.

**Figure 2:**
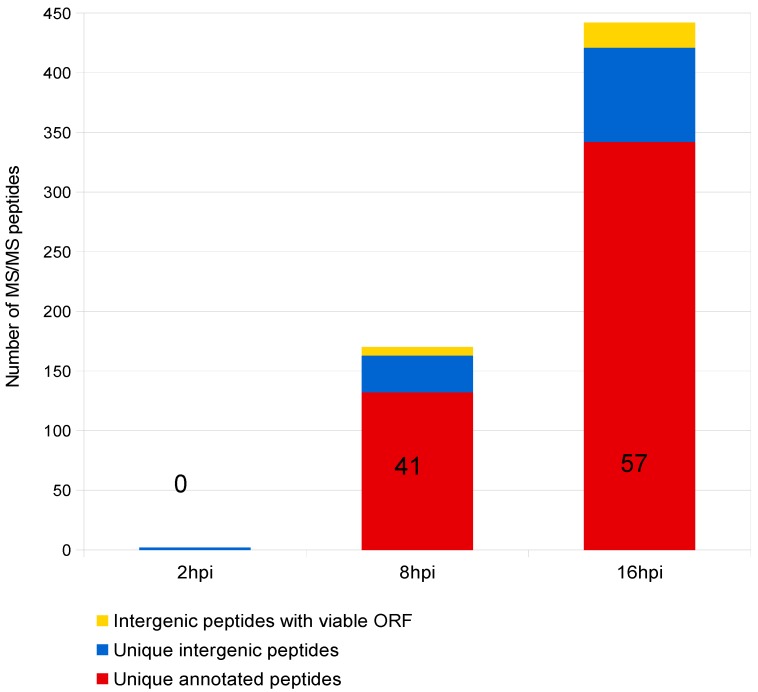
Quantification of unique BoHV-1 MS/MS spectra. Number of MS/MS spectra mapping to annotated viral ORFs (red) vs un-annotated genomic regions (blue). Yellow represents the number of spectra that mapped to un-annotated genomic regions that also contain nearby stop and start codons. The term unique refers to any peptide that maps to a single location of the genome. The bar graph is additive and not absolute. Numbers 0, 41, and 57 atop each bar represent the number of known viral proteins successfully identified.

After preparing infected cells for tandem mass spectrometry (MS/MS), the retrieved spectra were searched against a database consisting of the full genomic sequence of the BoHV-1 Cooper strain translated in all 6 reading frames. BoHV-1.1 proteogenomic mapping analysis identified a total of 2493 MS-MS spectra, 344 of them being unique (i.e., mapping to a single location in the whole BoHV-1.1 genome) and mapping to BoHV-1 currently annotated protein coding ORFs (Figure 1 and Figure 2; Table 2). In addition to these, other MS spectra mapped to viral genomic regions with no current annotation. We designated these *intergenic spectra* or *intergenic peptides.* We identified a total of 92 unique intergenic peptides (Figure 1 and Figure 2; Table 2). In order to determine if these peptides were encompassed within a potential ORF, the surrounding sequences were scanned up- and downstream in search of *in-frame* start and stop codons that could correspond to the putative new ORF. Out of the 92 unique intergenic peptides, 21 of them exhibited a potential ORF around them. Supplementary Figure S1 shows a diagram illustrating the proteogenomic mapping process and the work flow in this study. Peptides mapping to a region with no identifiable start and stop codons were excluded from further analysis. Table S1 summarizes the proteogenomic mapping data.

### 3.2. Validation of Intergenic Peptides with Surrounding Viable ORFs

**Figure 3:**
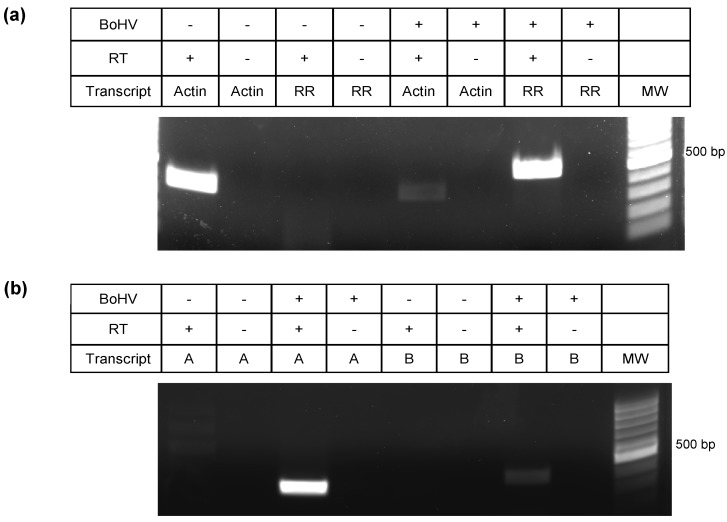
RT-PCR for two intergenic sequences. (**a**) PCR for cellular gene actin and viral gene ribonucleotide reductase (RR) on mock and cells infected for 16 h. (**b**) PCR amplification was carried out using primers for putative *ORF-A* and *ORF-B* for the same mock and infected cells. RT indicates the presence (+) or absence (-) of a retrotranscriptase step with mRNA as a template.

To determine whether RNA transcripts were being synthesized from the 21 putative new ORFs (intergenic peptides embedded within a predicted ORF), we used strand specific RT-PCR (Figure 3). Custom primers are shown in Table 1. The use of strand specific reverse transcription was imperative in this procedure to avoid the misleading amplification of the genes located directly antisense from the predicted sequences on the reverse strand (see Figure 1). For the intergenic transcripts shown in Figure 3b, *ORF-A* is located antisense to the *UL50* gene (encodes a dUTPase), and *ORF-B* is antisense to *UL46* (encodes a viral tegument protein). A negative control without retrotranscription is also imperative as a control of no genomic DNA contamination, or random cDNA synthesis from transcripts derived from the opposite strand. In none of our experiments did we obtain amplification of RT(-) samples (Figures 3, 5, and 8). The cellular gene for actin and the viral *bICP4* gene were used as positive controls to establish that samples were infected (+) or uninfected (-) (Figure 3a), while the successful retro-transcription and PCR amplification of two putative new transcripts, called *ORF-A* and *ORF-B*, at the predicted sizes is shown in Figure 3b. Of the 19 transcripts that were screened, 12 showed successful amplification. Two of the intergenic peptides were not screened by RT-PCR due to difficulties in primer design, since BoHV-1.1 has a 72% GC content. Importantly, amplification of putative new ORFs was never detected in mRNA derived from uninfected cells.

### 3.3. Characterization of 5′ Terminus of ORF-A via Rapid Amplification of cDNA Ends (RACE)

**Figure 4:**
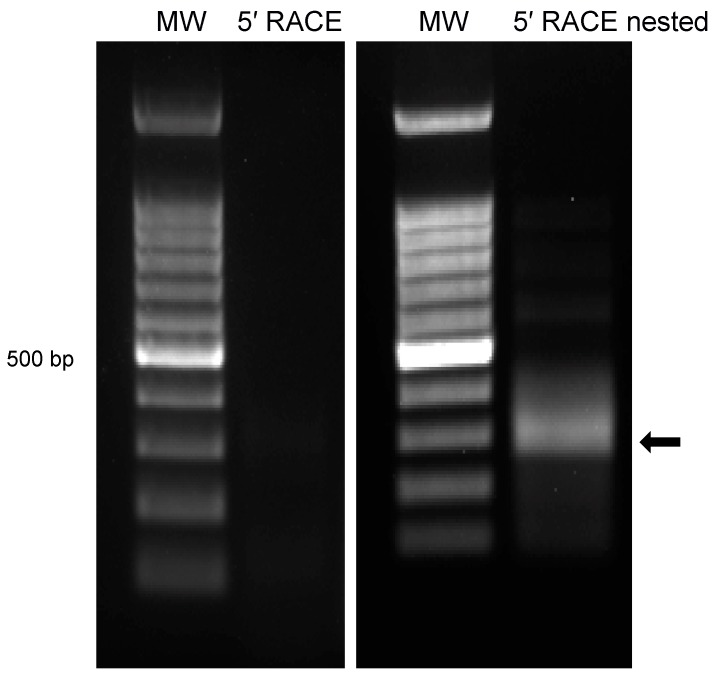
5′ RACE product for *ORF-A*. Infected cells were subjected to mRNA isolation followed by RACE protocol (Invitrogen GeneRacer) to map the 5′ terminus of the ORF A transcript. After the initial primers lead to poor PCR amplification (**left**), a nested PCR (**right**) was performed using the initial RACE product as a template. The 300 bp amplicon (black arrow) was excised and sent for sequencing.

In order to obtain the full-length sequence of the predicted ORFs, RACE was used to amplify the 5′ extension of the transcript. This was achieved using a combination of primers that align with the predicted coding region and a specialized primer provided in the Invitrogen kit that binds to the 5′ cap structure of mRNA transcripts. Nested primers were then used with the initial PCR product as template for increased specificity. The final PCR result showed a significant amplicon around 300 kb for the 5′ RACE product (Figure 4). The amplicon was excised, sequenced and blasted to align with genomic sequences consistent with the predicted location of *ORF-A*, with its 5′ boundary at nucleotide 7947 on the forward strand of the BoHV-1.1 genome (Figure 6).

### 3.4. Characterization of 3′ Terminus of the A Transcript by Primer Walking

**Figure 5:**
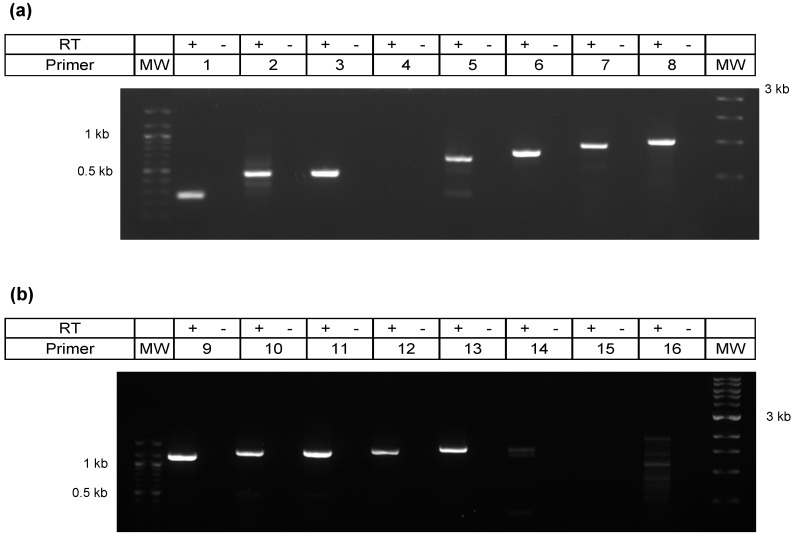
Primer walking for elucidation of *ORF-A* 3′ terminus. Strand-specific RT-PCR of BoHV-1.1-infected cell mRNA extracts was was performed on cDNA produced from BoHV-1 infected cells using 16 different reverse primers that anneal further down (3′) to the viral genome. Successful amplification allowed for capture of increasing lengths of the *ORF-A* transcript sequence. RT indicates the presence (+) or absence (-) of a retrotranscriptase step with mRNA as a template. (**a**) Amplicons were produced with primers 1–8 using a 1-min extension time during PCR. (**b**) Amplicons were produced using primers 9-16 with a 2-min extension time to account for the increase in amplicon length.

**Figure 6:**
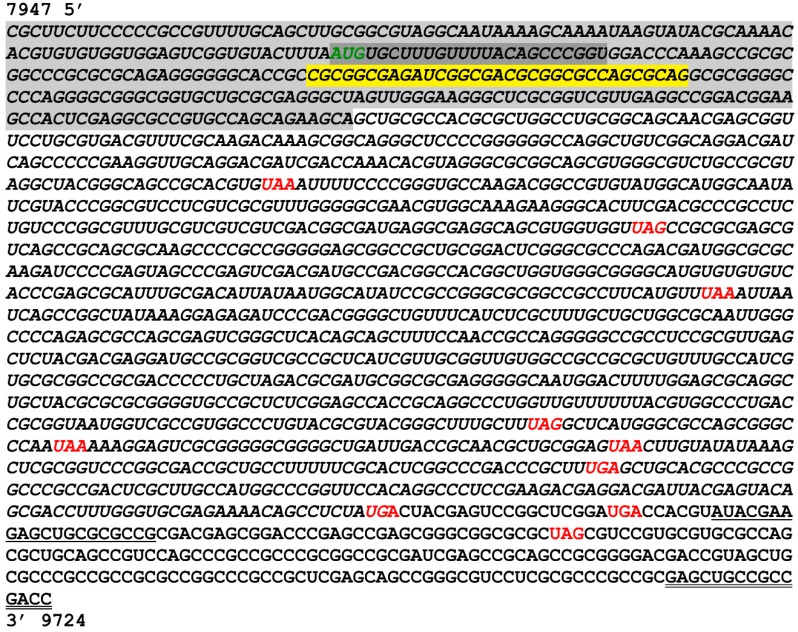
*ORF-A* Transcript Sequence. Sequence for *ORF-A* as determined by 5′ RACE and 3′ primer walking. The sequenced 5′ RACE product is shaded light gray and the darker gray shading indicates the location of the forward primer used for the 3′ walking strategy. Yellow highlighted text shows the sequence that encodes the experimental peptide found via MS/MS. Potential canonical start and stop codons are indicated in green and red, respectively. Italic text is the region of the transcript that has been confirmed by sequencing. Single and double underlined text show the binding site for primers 13 and 14, respectively.

Characterizing the 3′ end of *ORF-A* transcript via RACE yielded suboptimal results, leading us to adopt an alternative strategy. Sixteen reverse primers were designed that progressively covered the transcript to its 3′ terminus. These *strand-specific* reverse primers were used for individual cDNA synthesis reactions to avoid retro-transcription of transcripts generated from the negative strand. After PCR, successful and increasingly longer amplicons were obtained (Figure 5). Primer 13 produced the longest amplicon, which was 1582 bp (Figure 5). Downstream of this location (primers 14–16) did not yield consistent amplification. Therefore, we predict the 3′ end of *ORF-A* transcript to fall after nucleotide 9529 but before nucleotide 9724 on the forward strand of the BoHV-1.1 genome (Figure 6). Amplicons were sequenced and blasted against the BoHV-1.1 genome to confirm the identity of the viral sequence.

### 3.5. The Novel ORF-A Expresses a 55 KDa Protein in Bovine Cells

**Figure 7:**
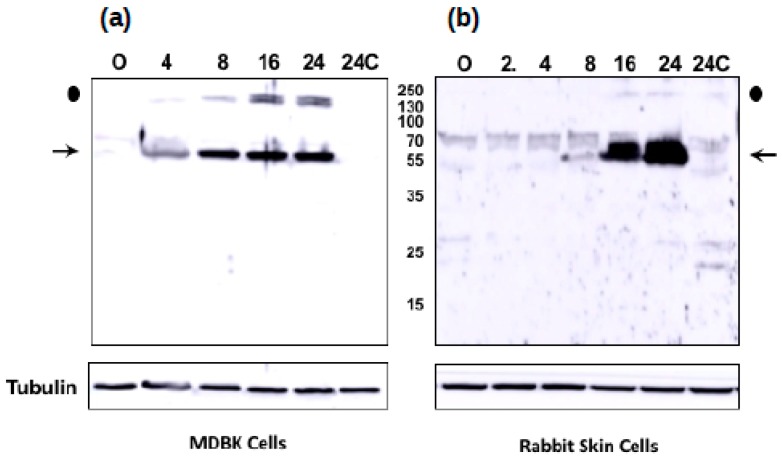
Immunoblot for ORF-A protein production. MDBK (panel **a**) or RS cells (panel **b**) were infected with BoHV-1 at the indicated times after infection (hours). Proteins were separated via SDS-PAGE gel (50 ug protein/lane). Western blots were probed using the ORF-A peptide specific antiserum. Tubulin was used as a protein loading control. These results are the average of three independent experiments. A protein around 55 kDa was detected in both infected cell types. Peptide-speific antisera from two rabbits yielded similar results in both cell lines. Data from only one antiserum is shown here.

To test whether ORF-A expresses a protein during infection, wild-type BoHV-1 was used to infect bovine kidney (MDBK) or rabbit skin cells (RS). At various times after infection, whole cell lysate was prepared, and Western blot analysis performed after separation of proteins on an SDS polyacrylamide gel. By 4 h after infection of MDBK cells, a viral specific protein migrating at approximately 55 kDa was detected (Figure 7; denoted by arrow). The size of the protein is consistent with a mRNA transcript of about 1582 bp (Figure 5). The intensity of this protein increased at later times after infection. Control uninfected cells (0 and 24 h after infection) were not specifically recognized by the peptide-specific antibody generated against ORF-A. Tubulin was included as a protein loading control. An additional viral-specific protein was detected that was migrating at approximately 140 kDa (Figure 7, denoted by closed circle). In RS cells, the peptide-specific ORF-A antiserum detected a viral specific 55 kDa protein at 16 and 24 h after infection: however, the 140 kDa viral protein was not readily detected. In general, BoHV-1 replication was slower, and the viral yields are lower in RS cells relative to MDBK cells. The purified peptide-specific antiserum from two different rabbits yielded similar results in Western blot studies in both cell lines (data not shown). In summary, this study provided evidence that ORF-A protein is expressed during productive infection.

### 3.6. ORF-A Transcription Profile is Consistent with Protein Synthesis

**Figure 8:**
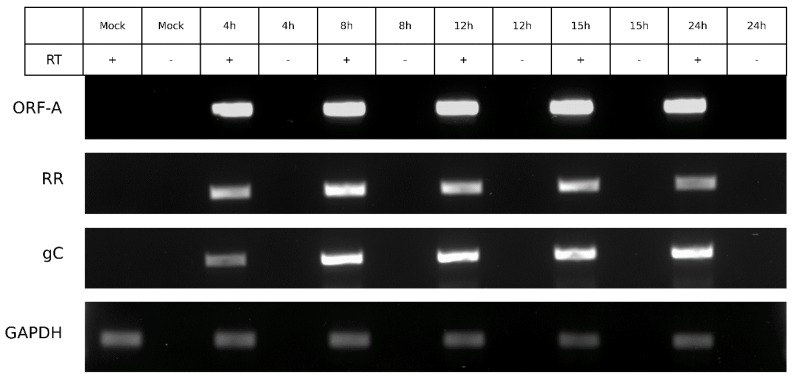
*ORF-A* is transcribed consistently throughout infection. CRIB cells infected with BoHV-1 over five infection time points (in hours, indicated at the top), followed by RNA extraction and gene specific RT-PCR. *ORF-A* transcript expression at the top, along with early viral gene ribonucleotide reductase (*RR*), late viral gene glycoprotein C (*gC*) and cellular gene *GAPDH*. Mock, uninfected cells.

To observe if the transcript expression aligned with ORF-A protein expression, strand-specific RT-PCR was carried out with infected cells at similar time points to the protein expression experiment (Figure 8). The expression of *ORF-A* transcript was detectable at 4 hpi and fairly consistent throughout infection. Viral genes ribonucleotide reductase (*RR*) and glycoprotein C (*gC*) were also amplified as controls of early and late viral gene expression, respectively. *GAPDH* was used as a housekeeping control. Overall, the expression of RNA is in agreement with the observed protein expression (Figure 7).

## 4. Discussion

Mass spectrometry has allowed for numerous opportunities in the annotation of genomes in cases of not only eukaryotes, but also bacteria and viruses [26,40,41,42]. The original BoHV-1 structural annotation based on computational gene prediction methods is more than two decades old and originated from multiple strains or subtypes of the virus. Experimental approaches, such as proteogenomics adopted in this study allow for increased accuracy in the characterization of the protein content produced during viral infection, for instance to correct gene boundaries and identify novel ORFs. By working backwards from protein to RNA, we were able to amplify the transcripts that encode potential new proteins, providing further evidence for their existence and paving the way for opportunities to associate previously unaccounted viral functions to new protein(s). In the case of *ORF-A*, we were able to characterize the full transcript and verify the expression of ORF A protein product via Western blot.

The finding of 92 intergenic peptides was encouraging, and 21 of these peptides are embedded within a viable ORF. The remaining peptides may have no canonical start or stop in-frame codons in adjacent sequences. In other cases, peptides map to a complex area of viral genes in close proximity in the same strand but different frame. A likely explanation for the existence of these scattered but proximal peptides is splicing, ribosomal frame-shifting of certain viral mRNA during translation, or perhaps aberrant transcripts during late stages of infection. BoHV-1 exhibits complex alternative patterns of splicing in certain genes (*LR* gene, *bICP0*, *bICP4* and *bICP22,* for example) [43,44,45]. As a result, one would not readily be able to predict an ORF in a given reading frame where an intergenic peptide mapped to. Alternatively, other initiation codons could be driving the synthesis of protein. Viruses have the potential to use alternative start codons and shift frames during transcription, which can increase the overall transcriptional capability [46,47]. Gene prediction algorithms scan for uninterrupted ORFs and search for traditional start and stop codons to predict potential ORFs, ignoring stop codon alternatives that have been noted in other organisms like UUG, CUU, or CCU [46,48,49]. In addition a plethora of codon usage alternatives have been documented in varied organisms, including several alternative start codons (https://www.ncbi.nlm.nih.gov/Taxonomy/Utils/wprintgc.cgi?mode=c). All these factors could confound gene prediction algorithms that missed these 21 ORFs.

It is also noteworthy that recent studies using deep sequencing of infected-cells transcriptomes have found a complex coding landscape for many herpesviruses [50], including long transcripts that were not known to exist. The transcriptome analysis of BoHV-1 would be a perfect future complement to our work and would elucidate some of the questions related to splicing and use of alternative start and stop codons.

Mapping the 5′ terminus of ORF-A transcript via RACE produced immediate and robust results indicating an unequivocal 5′ terminus for ORF-A transcript. However, RACE proved sub-optimal for mapping the 3′ terminus, leading us to use the primer walking strategy. This may be explained, in part, due to the exceptionally high GC content of the viral genome which interferes with PCR and may complicate primer design. In fact, two of the 21 intergenic peptides were not screened by strand specific RT-PCR because of difficulties in primer design. In the primer walking strategy, only one forward primer (located as upstream as possible) was used to avoid the amplification of downstream genes *UL49* and *UL49.5* that may or may not start transcription upstream, thus overlapping the *ORF-A* transcript. Therefore, we can only be certain of the existence of a transcript up to nucleotide 9529 (primer 13, Figure 5). The ORF-A transcript was successfully amplified with primers 1–3 and 5–13 (Table 1, Figure 5). Primer 13 yielded the longest amplicon of 1582 bp. If we assume the average molecular weight of an amino acid to be 110 Da, it can be predicted that a transcript of 1582 bp could direct the synthesis of a 50–55 kDa protein. This is an estimate, as we did not determine the transcription or the translation start sites of ORF-A. Further studies are needed to determine precise start and stop sites.

The viable ORF-A predicted around the MS/MS peptide was originally predicted to produce a protein of 15 kDa. After mapping the ORF-A transcript, we found that the transcript is at least 1582 nucleotides long (Figure 4 and Figure 5). Our result is consistent with the size of the ORF-A protein which is around 55 kDa as detected by Western blot analysis in both MDBK and RS cells (Figure 7). The original in silico-predicted ORF was smaller due to the existence of several stop codons that are in frame with the ORF-A MS/MS peptide. We know that splicing does not likely occur because the sequencing results show intact ampicons (Figure 6) that blasted back to the viral genome with no gaps (data not shown). While stop codon read-through is common in viral transcription [51,52,53], 9 in-frame stop codons may seem excessive, even if some of the stop codons localized to the 3′ untranslated region of the transcript. However, studies have shown potential for transcripts being produced by the readthrough of multiple stop codons in *Drosophila melanogaster* and *Anopheles gambiae* [54]. While the situation seems rare, this could be the case for ORF-A. Alternatively, if the ORF-A was smaller (with only a few readthrough events), the presence of post translational modifications such as phosphorylation or glycosylation on the protein could explain an apparently larger protein. Lastly, another common alternative in viral translation is ribosomal frameshifting [55,56]. However, this fact may not explain our results since stop codons also appear in the other two reading frames. Future work will likely elucidate the translational mechanism behind the synthesis of the ORF-A protein.

In conclusion, we have identified 21 intergenic peptides predicted to have a traditional ORF around them. The proof that 12 of them have an RNA associated is encouraging. In this study we further characterize one of these genes, which we have termed ORF-A. There are an additional 11 intergenic peptides that await discovery. Our results highlight the value of incorporating experimental data into gene discovery and annotation. By discovering and characterizing new genes, we expect to contribute to a more accurate BoHV-1 genome annotation, which could, in turn, provide insight to the field of vaccinology.

## Figures and Tables

**Table 1:** (**a**) Table of primers used for PCR amplification. (**b**) Reverse primers used for strand-specific retrotranscription during the 3′ walk strategy outlined in the text and in Figure 5.

(a)		
Target	Forward	Reverse
**A**	ATGTGCTTTGTTTTACAGCCCGGT	ACGTGCGGCTGCCCCGTAG
**B**	ATGGCCGCGATCATGTACGGGT	CGCTGGCTGCTGTGGTTCATGGA
**Actin**	CGTGACATTAAGGAGAAGCTGTGC	CTCAGGAGGAGCAATGATCTTGAT
**GAPDH**	CCATGGAGAAGGCTGGGG	CAAAGTTGTCATGGATGACC
**RR**	TTTTACGAGACCGAGTGCCC	GACGAAAAGGTTGTGGGTGC
**gC**	TGATCGCAGCTATTTTCGCC	TTCTGGGCTACGAACAGCAG
**5′ RACE**	provided by Invitrogen	CGTGGCGCAGCTGCTTCTGCTGG
**5′ Nested RACE**	provided by Invitrogen	GCTGTCAACGATACGCTACGTAACG
**(b)**		
**Reverse Primer**		
1	GCGAAACGTCACGCAGGA	
2	GCCCTACGTGTTTGGTCGAT	
3	GAGGACGCCGGGTACGATA	
4	ACGCTCGCGCGGCTAA	
5	GCTACTCGGGGATCTTGCG	
6	CCCGGCGGATATGCCATTA	
7	GTGAGCCCGACTCGCTG	
8	CGCAACGATGAGCGGCG	
9	CAGGGCCACGGCGACCATTA	
10	GCCGGGACCGCGAGCTT	
11	CGGGCCGAGTGCGAAAAAGG	
12	TTCGGAGGGCCTGTGGAACC	
13	CGGCGCGCAGCTCTTCGTAT	
14	GGTCGGCGGCAGCTC	
15	AGACGCAACAGCATTAGCACCGGGGG	
16	GACGGCCTCGCAAAAGATCGTTCGGT	

**Table 2:** Summary of proteogenomic mapping results.

	2 hpi	8 hpi	16 hpi	Total ^b^
Total viral peptides	4	498	1991	2493
Total annotated peptides	0	394	1662	2056
Unique ^a^ annotated peptides	0	132	342	344
Viral genes identified	0	41	57	57
Total intergenic peptides	4	104	329	437
Unique^a^ intergenic peptides	2	31	79	92
Intergenic peptides with viable ORF	0	7	21	21

^a^ unique = mapping to a single location on the BoHV-1 genome. ^b^ correcting for peptides that appeared in more than one timepoint. hpi, hours poist infection.

## Supplementary Materials

The following are available online at http://www.mdpi.com/1999-4915/10/9/499/s1, Figure S1: Diagram representing the proteogenomic mapping process. Table S1: Genomic coordinates and abundance of viral peptides mapped by the proteogenomic tool.
