# Supplementary material for: Proteogenomic Identification of a Novel Protein-Encoding Gene in Bovine Herpesvirus 1 That Is Expressed during Productive Infection
Source: Viruses. 2018 Sep 14;10(9):499. doi: 10.3390/v10090499 (PMC6164122; doi:10.3390/v10090499)
Supplement: Supplementary file 1 [file viruses-10-00499-s001.zip › viruses-338366-supplementary-Figure 1.pdf]

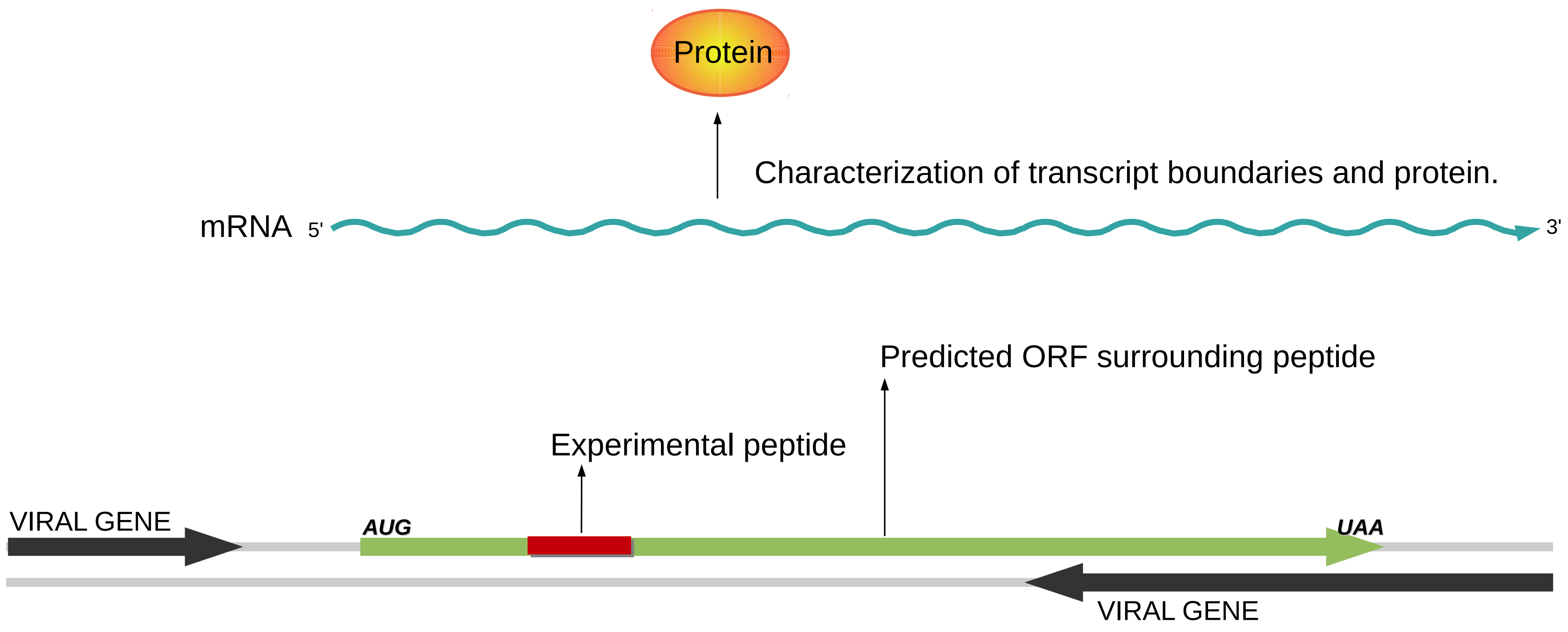

Supplementary Figure 1. Diagram representing the proteogenomic mapping process. The double stranded DNA viral genome (light gray parallel lines) with currently annotated genes (black arrows) on both strands. The green arrow represents a computationally predicted open reading frame (ORF) surrounding the genomic region where the experimental mass-spectrometry peptide mapped to (red), with corresponding start (AUG) and stop (UAA is depicted; could also be UAG and UGA) codons. The full extension of the mRNA transcript for the putative new gene (wavy teal line) is characterized to both 3’ and 5’ termini. Finally, a specific protein is detected in infected but not uninfected cells via western blot analysis using a peptide-specific antibody.
