# Supplementary material for: Proteogenomic Identification of a Novel Protein-Encoding Gene in Bovine Herpesvirus 1 That Is Expressed during Productive Infection
Source: Viruses. 2018 Sep 14;10(9):499. doi: 10.3390/v10090499 (PMC6164122; doi:10.3390/v10090499)
Supplement: Supplementary file 1 [file viruses-10-00499-s001.zip › viruses-338366-supplementary-Table 1.pdf]

Supplemental table 1. Genomic coordinates and abundance of viral peptides mapped by the proteogenomic tool. Predicted ORF boundaries are provided for those intergenic peptides that were predicted to have a viable ORF around them.

Peptide Start	Peptide End	Strand	Corresponding Gene[a]	Abundance	Predicted Start Codon[b]	Predicted Stop Codon[b]
652	687	F	CIRC	2	-	-
733	750	F	CIRC	1	-	-
736	780	F	CIRC	8	-	-
1675	1719	R	UL54	4	-	-
2011	2061	R	UL54	7	-	-
2224	2259	R	UL54	3	-	-
3518	3610	R	UL53	2	-	-
3749	3811	R	UL54	1	-	-
4143	4178	R	UL52	3	-	-
6582	6614	R	UL52	1	-	-
8265	8306	R	UL50	3	-	-
8385	8426	R	UL50	1	-	-
8385	8441	R	UL50	5	-	-
8442	8519	R	UL50	8	-	-
8697	8774	R	UL50	13	-	-
8772	8819	R	UL50	1	-	-
8841	8882	R	UL50	10	-	-
9049	9075	F	UL49.5	8	-	-
9055	9075	F	UL49.5	2	-	-
9076	9123	F	UL49.5	2	-	-
9079	9123	F	UL49.5	2	-	-
9226	9255	F	UL49.5	5	-	-
9409	9465	F	UL49	12	-	-
9802	9864	F	UL49	1	-	-
10693	10785	F	UL48	3	-	-
10978	11013	F	UL48	4	-	-
11299	11328	F	UL48	1	-	-
11503	11547	F	UL48	10	-	-
12093	12125	F	UL47	1	-	-
12264	12317	F	UL47	2	-	-
12264	12365	F	UL47	2	-	-
12306	12365	F	UL47	3	-	-
12318	12365	F	UL47	10	-	-
12366	12401	F	UL47	18	-	-
12585	12626	F	UL47	5	-	-
12636	12695	F	UL47	3	-	-
12696	12761	F	UL47	4	-	-
13077	13121	F	UL47	6	-	-
13179	13262	F	UL47	19	-	-
13179	13265	F	UL47	1	-	-
13182	13262	F	UL47	13	-	-
13227	13262	F	UL47	1	-	-
13263	13301	F	UL47	8	-	-
13338	13424	F	UL47	60	-	-
13482	13541	F	UL47	2	-	-

Peptide Start	Peptide End	Strand	Corresponding Gene[a]	Abundance	Predicted Start Codon[b]	Predicted Stop Codon[b]
13698	13745	F	UL47	25	-	-
13758	13802	F	UL47	3	-	-
13899	13937	F	UL47	2	-	-
13899	13970	F	UL47	14	-	-
13938	13970	F	UL47	6	-	-
13995	14030	F	UL47	28	-	-
13995	14033	F	UL47	2	-	-
14037	14087	F	UL47	1	-	-
14088	14180	F	UL47	10	-	-
14106	14180	F	UL47	4	-	-
14562	14597	F	UL46	1	-	-
14610	14651	F	UL46	1	-	-
14886	14945	F	UL46	16	-	-
14913	14945	F	UL46	4	-	-
15348	15386	F	UL46	2	-	-
15399	15464	F	UL46	6	-	-
17093	17140	R	UL44	4	-	-
17183	17239	R	UL44	42	-	-
17240	17278	R	UL44	1	-	-
17426	17449	R	UL44	8	-	-
17480	17518	R	UL44	2	-	-
17540	17590	R	UL44	3	-	-
17642	17677	R	UL44	3	-	-
18472	18495	R	UL43	3	-	-
19708	19770	R	UL42	6	-	-
19708	19767	R	UL42	50	-	-
19708	19740	R	UL42	2	-	-
20113	20178	R	UL42	9	-	-
20236	20286	R	UL42	13	-	-
20236	20268	R	UL42	3	-	-
20287	20331	R	UL42	18	-	-
20302	20331	R	UL42	1	-	-
20332	20370	R	UL42	20	-	-
20407	20481	R	UL42	1	-	-
20497	20520	R	UL42	2	-	-
20521	20550	R	UL42	6	-	-
20563	20592	R	UL42	1	-	-
20593	20658	R	UL42	10	-	-
20659	20682	R	UL42	2	-	-
20683	20733	R	UL42	8	-	-
20734	20799	R	UL42	3	-	-
20821	20847	R	UL42	1	-	-
22061	22099	F	UL41	17	-	-
22211	22237	F	UL41	7	-	-
22265	22300	F	UL41	1	-	-
22737	22796	R	UL40	6	-	-
23118	23159	R	UL40	1	-	-
23178	23210	R	UL40	3	-	-
23211	23264	R	UL40	4	-	-
23376	23426	R	UL40	3	-	-
23821	23892	R	UL39	5	-	-

Peptide Start	Peptide End	Strand	Corresponding Gene[a]	Abundance	Predicted Start Codon[b]	Predicted Stop Codon[b]
23821	23865	R	UL39	1	-	-
23821	23880	R	UL39	2	-	-
23944	23991	R	UL39	3	-	-
24277	24303	R	UL39	2	-	-
24523	24642	R	UL39	1	-	-
24709	24765	R	UL39	5	-	-
24775	24810	R	UL39	3	-	-
24862	24885	R	UL39	2	-	-
25474	25524	R	UL39	6	-	-
25597	25662	R	UL39	5	-	-
26361	26390	R	UL38	5	-	-
26421	26453	R	UL38	1	-	-
26616	26663	R	UL38	5	-	-
27006	27038	R	UL38	1	-	-
27009	27038	R	UL38	2	-	-
27201	27251	R	UL38	5	-	-
27330	27395	R	UL38	2	-	-
28094	28153	F	UL37	14	-	-
28394	28441	F	UL37	2	-	-
28448	28513	F	UL37	1	-	-
28862	28897	F	UL37	12	-	-
30425	30457	F	UL37	2	-	-
30491	30535	F	UL37	8	-	-
30578	30628	F	UL37	2	-	-
31626	31664	F	UL36	5	-	-
31689	31754	F	UL36	6	-	-
32373	32429	F	UL36	3	-	-
33363	33461	F	UL36	15	-	-
34131	34157	F	UL36	5	-	-
34929	34988	F	UL36	2	-	-
35247	35276	F	UL36	2	-	-
35415	35468	F	UL36	3	-	-
35841	35873	F	UL36	3	-	-
35961	35996	F	UL36	1	-	-
36297	36371	F	UL36	5	-	-
37599	37691	F	UL36	1	-	-
37599	37634	F	UL36	7	-	-
39381	39491	F	UL36	1	-	-
41152	41196	R	UL35	1	-	-
41197	41241	R	UL35	1	-	-
41197	41289	R	UL35	1	-	-
41242	41289	R	UL35	2	-	-
41484	41549	R	UL34	6	-	-
41643	41669	R	UL34	6	-	-
43359	43403	F	UL32	6	-	-
44707	44748	F	UL31	2	-	-
44923	44976	F	UL31	4	-	-
45067	45123	F	UL31	9	-	-
47270	47305	R	UL30	2	-	-
48179	48208	R	UL30	5	-	-
48635	48694	R	UL30	1	-	-

Peptide Start	Peptide End	Strand	Corresponding Gene[a]	Abundance	Predicted Start Codon[b]	Predicted Stop Codon[b]
49142	49177	R	UL30	1	-	-
49516	49554	F	UL29	1	-	-
49648	49701	F	UL29	2	-	-
49834	49893	F	UL29	1	-	-
50485	50514	F	UL29	1	-	-
50515	50604	F	UL29	5	-	-
50545	50604	F	UL29	1	-	-
50605	50631	F	UL29	11	-	-
50632	50673	F	UL29	2	-	-
50707	50724	F	UL29	6	-	-
50806	50826	F	UL29	1	-	-
51184	51225	F	UL29	44	-	-
51226	51252	F	UL29	7	-	-
51253	51309	F	UL29	7	-	-
51310	51336	F	UL29	2	-	-
51436	51516	F	UL29	1	-	-
51550	51591	F	UL29	2	-	-
51736	51780	F	UL29	1	-	-
51787	51870	F	UL29	12	-	-
51883	51957	F	UL29	8	-	-
51976	52017	F	UL29	4	-	-
51976	52020	F	UL29	5	-	-
51988	52020	F	UL29	5	-	-
52186	52215	F	UL29	5	-	-
52270	52329	F	UL29	1	-	-
52330	52380	F	UL29	4	-	-
52744	52797	F	UL29	18	-	-
52939	52983	F	UL29	4	-	-
52939	52986	F	UL29	3	-	-
56167	56211	F	UL27	8	-	-
56254	56313	F	UL27	8	-	-
56257	56313	F	UL27	1	-	-
56335	56391	F	UL27	16	-	-
56554	56580	F	UL27	3	-	-
56554	56583	F	UL27	2	-	-
56638	56676	F	UL27	15	-	-
56935	56985	F	UL27	8	-	-
57151	57237	F	UL27	3	-	-
57409	57432	F	UL27	3	-	-
57589	57633	F	UL27	4	-	-
57706	57735	F	UL27	1	-	-
57736	57783	F	UL27	1	-	-
57787	57807	F	UL27	3	-	-
59084	59101	R	UL26.5	6	-	-
59198	59245	R	UL26.5	2	-	-
59372	59392	R	UL26.5	2	-	-
59372	59395	R	UL26.5	1	-	-
59417	59470	R	UL26.5	25	-	-
59417	59467	R	UL26.5	10	-	-
59420	59467	R	UL26.5	1	-	-
60026	60079	R	UL26	1	-	-

Peptide Start	Peptide End	Strand	Corresponding Gene[a]	Abundance	Predicted Start Codon[b]	Predicted Stop Codon[b]
61023	61112	R	UL25	1	-	-
61569	61610	R	UL25	5	-	-
61665	61694	R	UL25	1	-	-
62511	62537	R	UL25	2	-	-
63191	63208	R	UL24	1	-	-
63471	63503	F	UL23	10	-	-
63738	63770	F	UL23	2	-	-
64750	64815	F	UL22	6	-	-
64870	64911	F	UL22	2	-	-
65059	65091	F	UL22	2	-	-
65059	65124	F	UL22	1	-	-
65188	65253	F	UL22	5	-	-
65272	65331	F	UL22	3	-	-
65278	65331	F	UL22	6	-	-
65560	65589	F	UL22	9	-	-
65692	65742	F	UL22	7	-	-
65695	65742	F	UL22	5	-	-
65941	66006	F	UL22	20	-	-
66067	66114	F	UL22	33	-	-
66373	66429	F	UL22	8	-	-
66661	66726	F	UL22	11	-	-
66829	66879	F	UL22	2	-	-
66877	66900	F	UL22	2	-	-
67470	67547	R	UL21	11	-	-
67548	67598	R	UL21	3	-	-
67617	67652	R	UL21	2	-	-
68124	68168	R	UL21	5	-	-
68283	68318	R	UL21	2	-	-
68352	68390	R	UL21	5	-	-
68481	68498	R	UL21	3	-	-
70170	70238	F	UL19	5	-	-
70239	70286	F	UL19	34	-	-
70569	70595	F	UL19	4	-	-
70599	70628	F	UL19	5	-	-
70737	70781	F	UL19	11	-	-
71007	71039	F	UL19	2	-	-
71040	71066	F	UL19	3	-	-
71088	71165	F	UL19	25	-	-
71175	71213	F	UL19	5	-	-
71445	71483	F	UL19	1	-	-
71445	71489	F	UL19	1	-	-
71748	71777	F	UL19	1	-	-
71778	71849	F	UL19	11	-	-
71790	71849	F	UL19	17	-	-
71793	71849	F	UL19	1	-	-
71814	71849	F	UL19	3	-	-
72009	72038	F	UL19	2	-	-
72210	72242	F	UL19	11	-	-
72243	72269	F	UL19	7	-	-
72309	72371	F	UL19	4	-	-
72372	72407	F	UL19	1	-	-

Peptide Start	Peptide End	Strand	Corresponding Gene[a]	Abundance	Predicted Start Codon[b]	Predicted Stop Codon[b]
72525	72587	F	UL19	2	-	-
72558	72587	F	UL19	1	-	-
72588	72650	F	UL19	14	-	-
72720	72770	F	UL19	12	-	-
72771	72863	F	UL19	2	-	-
72894	72947	F	UL19	1	-	-
72948	72980	F	UL19	32	-	-
73029	73061	F	UL19	8	-	-
73035	73061	F	UL19	2	-	-
73062	73100	F	UL19	7	-	-
73185	73229	F	UL19	7	-	-
73230	73286	F	UL19	3	-	-
73287	73337	F	UL19	2	-	-
73407	73475	F	UL19	25	-	-
73563	73655	F	UL19	6	-	-
73656	73685	F	UL19	3	-	-
73971	74018	F	UL19	1	-	-
74019	74054	F	UL19	19	-	-
74302	74388	F	UL18	2	-	-
74698	74727	F	UL18	1	-	-
75028	75063	F	UL18	3	-	-
77776	77808	F	UL17	1	-	-
77878	77970	F	UL17	5	-	-
78904	78945	F	UL16	2	-	-
79648	79680	F	UL16	1	-	-
84170	84205	F	UL12	8	-	-
84451	84564	F	UL11	3	-	-
84888	84932	R	UL10	6	-	-
84975	85013	R	UL10	19	-	-
84975	85010	R	UL10	13	-	-
86013	86048	R	UL10	3	-	-
87796	87837	R	UL9	1	-	-
87859	87954	R	UL9	2	-	-
91016	91042	R	UL7	1	-	-
91043	91069	R	UL7	6	-	-
91685	91738	R	UL7	1	-	-
92634	92693	R	UL6	1	-	-
92826	92855	R	UL6	1	-	-
93537	93566	R	UL6	2	-	-
94537	94566	F	UL5	4	-	-
95893	95931	F	UL5	1	-	-
97287	97331	R	UL3.5	2	-	-
98277	98318	R	UL2	2	-	-
98343	98399	R	UL2	10	-	-
98400	98423	R	UL2	2	-	-
98424	98465	R	UL2	9	-	-
98610	98645	R	UL2	9	-	-
98646	98681	R	UL2	6	-	-
98646	98669	R	UL2	1	-	-
99187	99240	R	UL1	21	-	-
99376	99447	R	UL1	8	-	-

Peptide Start	Peptide End	Strand	Corresponding Gene[a]	Abundance	Predicted Start Codon[b]	Predicted Stop Codon[b]
99454	99489	R	UL1	3	-	-
102713	102730	R	BICP0	4	-	-
104444	104518	R	BICP4	2	-	-
104798	104836	R	BICP4	4	-	-
105005	105043	R	BICP4	12	-	-
105044	105085	R	BICP4	4	-	-
105272	105355	R	BICP4	4	-	-
112954	112992	F	BICP22	32	-	-
113044	113073	F	BICP22	2	-	-
114180	114209	R	US1.67	3	-	-
114324	114341	R	US1.67	2	-	-
114642	114725	R	US1.67	4	-	-
116226	116252	F	US3	8	-	-
116253	116303	F	US3	5	-	-
116349	116375	F	US3	4	-	-
116460	116504	F	US3	7	-	-
116517	116540	F	US3	2	-	-
116889	116933	F	US3	2	-	-
117033	117065	F	US3	3	-	-
117066	117122	F	US3	1	-	-
117374	117442	F	US4	24	-	-
117854	117880	F	US4	7	-	-
118954	118998	F	US6	23	-	-
119497	119538	F	US6	7	-	-
119539	119565	F	US6	1	-	-
119566	119604	F	US6	24	-	-
119581	119604	F	US6	5	-	-
120303	120359	F	US7	5	-	-
120366	120401	F	US7	8	-	-
120558	120611	F	US7	3	-	-
120561	120611	F	US7	2	-	-
120642	120677	F	US7	5	-	-
120684	120734	F	US7	1	-	-
121272	121325	F	US7	3	-	-
121721	121777	F	US8	6	-	-
121778	121828	F	US8	2	-	-
121955	122056	F	US8	4	-	-
122309	122368	F	US8	8	-	-
122933	122950	F	US8	5	-	-
123260	123304	F	US8	3	-	-
123275	123304	F	US8	5	-	-
123305	123343	F	US8	2	-	-
123668	123685	F	US9	1	-	-
8115	8147	F	-	1	8049	8450
15305	15355	F	-	1	15382	14981
19752	19769	R	-	6	19841	19530
19781	19798	F	-	1	19751	19948
22048	22095	R	-	1	22380	21721
27034	27072	F	-	4	27004	27336
31841	31858	R	-	1	31811	34429
38345	38380	R	-	1	40789	38201

Peptide Start	Peptide End	Strand	Corresponding Gene[a]	Abundance	Predicted Start Codon[b]	Predicted Stop Codon[b]
51636	51686	R	-	1	51764	51069
53281	53298	R	-	12	53640	52267
59025	59132	F	-	1	58935	59183
63979	63996	R	-	2	63996	63979
64776	64793	R	-	1	64982	64512
73409	73504	R	-	1	73501	73268
92452	92469	F	-	12	92425	92652
94731	94751	R	-	24	95042	94689
97283	97312	F	-	1	97076	97423
99933	99950	F	-	60	99882	100163
105492	105515	F	-	2	105477	106508
116959	117021	R	-	1	117498	116950
117271	117309	R	-	2	117498	116950
118650	118679	R	-	1	118820	118587
1120	1179	R	-	1	-	-
4338	4355	F	-	1	-	-
9374	9391	F	-	1	-	-
10195	10230	F	-	1	-	-
12398	12415	F	-	20	-	-
22006	22047	F	-	1	-	-
24185	24217	R	-	2	-	-
25113	25154	R	-	1	-	-
26401	26427	R	-	6	-	-
27724	27768	F	-	1	-	-
29297	29314	R	-	2	-	-
29577	29594	F	-	6	-	-
30334	30351	R	-	1	-	-
30538	30573	F	-	1	-	-
34918	34935	R	-	12	-	-
36898	36915	F	-	1	-	-
37529	37546	R	-	1	-	-
40863	40895	R	-	1	-	-
41541	41576	F	-	1	-	-
41958	42002	F	-	2	-	-
42276	42293	F	-	4	-	-
45608	45625	F	-	1	-	-
46809	46826	F	-	1	-	-
51630	51671	F	-	1	-	-
60429	60443	R	-	8	-	-
60766	60819	F	-	2	-	-
65858	65908	F	-	1	-	-
67008	67058	F	-	2	-	-
68722	68739	F	-	1	-	-
68929	68946	R	-	18	-	-
74074	74106	R	-	1	-	-
77021	77074	F	-	2	-	-
78059	78091	F	-	1	-	-
78129	78173	R	-	1	-	-
79455	79469	F	-	32	-	-
80832	80846	F	-	33	-	-
83661	83678	R	-	5	-	-

Peptide Start	Peptide End	Strand	Corresponding Gene[a]	Abundance	Predicted Start Codon[b]	Predicted Stop Codon[b]
85505	85525	R	-	5	-	-
86232	86270	R	-	1	-	-
88036	88053	R	-	7	-	-
89021	89071	R	-	17	-	-
90690	90707	F	-	13	-	-
94646	94663	R	-	2	-	-
94662	94679	F	-	6	-	-
95134	95154	R	-	3	-	-
95993	96007	R	-	4	-	-
96276	96317	F	-	1	-	-
98898	98912	F	-	4	-	-
99815	99868	F	-	1	-	-
100215	100259	F	-	1	-	-
100746	100763	R	-	1	-	-
101612	101629	F	-	1	-	-
106339	106368	R	-	12	-	-
108679	108717	F	-	1	-	-
109188	109202	F	-	11	-	-
109620	109664	R	-	2	-	-
110271	110288	F	-	2	-	-
114069	114155	F	-	1	-	-
114082	114147	F	-	1	-	-
115430	115447	F	-	1	-	-
118498	118515	F	-	1	-	-
118742	118801	F	-	1	-	-
118883	118900	R	-	12	-	-
119509	119538	R	-	1	-	-
121058	121084	R	-	1	-	-
121326	121349	R	-	1	-	-
121372	121410	F	-	1	-	-
123747	123833	F	-	1	-	-
128860	128874	R	-	11	-	-

[a] Peptides without corresponding genes refer to those found in intergenic regions

[b] stop and start codon location around intergenic peptides predicted to be embedded in a viable ORF.
